# The Cervix in Ectopic Gestation

**Published:** 1904-06

**Authors:** 


### The Cervix in Ectopic Gestation.

Pinard, in giving his opinion on this subject, says he finds the modification of the cervix uteri in extrauterine pregnancy is not properly interpreted. In reports we read "Cervix small, firm, not softened." These words mislead many students and gynecologists. The cervix always becomes softened in pregnancy, whether normal or extrauterine, but it must not be forgotten that the softening process passes away when the ovum dies. Very often when ectopic gestation is diagnosticated that condition exists, clinically speaking, but the fetus has for some time been dead. The cervix in such a case is firm, for, obstetrically speaking, there is no pregnancy, nothing but a fetal sac. Pinard notes that when an extrauterine pregnancy has gone on to term, the cervix uteri may be so soft that an experienced obstetrician may suspect that the gestation must be normal after all.—*British Medical Journal.*

